# Knowledge, attitudes and practices among medical workers toward outpatient diabetes information platform

**DOI:** 10.1186/s12913-024-10711-y

**Published:** 2024-03-12

**Authors:** Yi Peng, Jianying Duan, Jian Hou, Nan Xu, Jiaming Wu, Xijing Bao, Qian Yao, Yang Li

**Affiliations:** 1 Department of Endocrinology, Zhangjiakou First Hospital, Zhangjiakou, 075000 China; 2 Department of Pharmacology, Zhangjiakou First Hospital, Zhangjiakou, 075000 China

**Keywords:** Knowledge, Attitudes, Practices, Diabetes information platform, Cross-sectional study

## Abstract

**Background:**

This study aimed to assess the knowledge, attitudes and practices among medical workers toward outpatient diabetes information platform.

**Methods:**

This web-based cross-sectional study was conducted between May 2023 and June 2023 at the First Hospital of Zhangjiakou, China. A self-designed questionnaire was developed to collect demographic information of medical workers, and assess their knowledge, attitudes and practices toward outpatient diabetes information platform.

**Results:**

A total of 685 questionnaires were collected. Among the participants, 603 (88.03%) were female, 432 (63.07%) work in a tertiary hospital, 548 (80.00%) have a bachelor degree, 270 (39.42%) of them work in the department of internal medicine and 315 (45.99%) of them received previous training on outpatient diabetes information platform. The mean knowledge, attitudes and practices scores were 4.32 ± 1.27 (possible range: 0–6), 56.76 ± 5.72 (possible range: 14–70), and 32.22 ± 8.42 (possible range: 9–45), respectively. 350 (51.09%) of them have sufficient knowledge, 168 (24.53%) have positive attitudes and 395 (57.66%) have active practices. Pearson correlation analysis showed that knowledge was positively correlated with attitudes (*r* = 0.397, *P <* 0.001), and attitudes were positively correlated with practices (*r* = 0.306, *P <* 0.001). Multivariate analysis showed that primary hospital (OR = 0.32, 95% CI: 0.14–0.71, *P =* 0.005), secondary hospital (OR = 0.48, 95% CI: 0.32–0.72, *P <* 0.001), doctor (OR = 2.44, 95% CI: 1.39–4.28, *P =* 0.002) were independently associated with sufficient knowledge. Knowledge (OR = 1.49, 95% CI: 1.29–1.73, *P <* 0.001), community hospital staff (OR = 0.21, 95% CI: 0.05–0.88, *P =* 0.032) were independently associated with positive attitudes. Attitudes (OR = 1.13, 95% CI: 1.09–1.17, *P <* 0.001), junior college (OR = 1.72, 95% CI: 1.07–2.77, *P =* 0.026) were independently associated with active practices. The structural equation model demonstrated that knowledge had a direct effect on attitudes (path coefficient = 0.521, *P <* 0.001), and attitudes had a direct effect on practices (path coefficient = 0.542, *P <* 0.001). Moreover, the type of hospital had a direct effect on knowledge (path coefficient = 0.085, *P <* 0.001). Additionally, previous training on the outpatient diabetes platform had direct effects on attitudes (path coefficient = 0.191, *P <* 0.001) and practices (path coefficient = 0.184, *P <* 0.001).

**Conclusion:**

These findings revealed that medical workers have insufficient knowledge, positive attitudes and inactive practices toward the outpatient diabetes information platform. Comprehensive training programs are needed to improve medical staff’s practices in this area.

**Supplementary Information:**

The online version contains supplementary material available at 10.1186/s12913-024-10711-y.

## Background

Diabetes mellitus is a chronic metabolic disorder characterized by elevated blood glucose levels resulting from impaired insulin secretion, insulin action, or both [1, 2]. China has witnessed a particularly rapid increase in the prevalence of diabetes [3]. The burden imposed by diabetes extends beyond financial implications and encompasses long-term complications such as cardiovascular diseases, renal failure, and retinopathy, which significantly impact individuals’ quality of life and life expectancy [4–6].

Recognizing the critical importance of effective diabetes management and control, medical systems have shifted their focus to outpatient management approaches. The concept of outpatient diabetes information platforms integrates electronic health records, telemedicine, mobile applications, and other technologies to provide comprehensive care, enhance patient self-management, and improve communication between medical providers and medical workers [7, 8]. In this context, the utilization of information technology and digital platforms for diabetes management has garnered significant attention [9, 10].

Compared to traditional outpatient diabetes management models, outpatient information platforms offer several advantages [11, 12]. They provide real-time access to patient data, enabling timely monitoring and adjustment of treatment plans. Meanwhile, these platforms empower medical workers to actively engage in self-care through the provision of educational resources, personalized guidance, and remote monitoring tools [13]. Moreover, they enhance communication and collaboration among medical professionals, fostering a coordinated and patient-centered approach to diabetes management [14]. Currently, there have been some preliminary studies investigating the advantages of utilizing diabetes information management platforms. In China, a randomized clinical trial was conducted to examine the effectiveness of using mobile phone SMS for information-driven glycemic control interventions in patients with coronary heart disease and diabetes mellitus. The research findings indicate that the text message intervention led to improved glycemic control in patients with diabetes mellitus and coronary heart disease [15]. Likewise, a study on the influence of information-based continuous care on disease control and treatment compliance of Chinese elderly diabetic patients reveals that information-based continuous care yields favorable effects on disease control and treatment compliance among elderly diabetic patients. It can assist in managing blood sugar levels and optimizing patients’ self-management capabilities, holding significant clinical value for broader implementation [16].

The efficacy of outpatient diabetes information platforms hinges significantly on healthcare professionals’ awareness and attitudes towards them [17]. In a study examining the utilization of a mobile health (mHealth) recommender system, enabled by a personal health library, for diabetes self-management in underserved communities, it was emphasized that healthcare professionals play a pivotal role in the efficacy of this remote system. This emphasis indicates that the system’s effectiveness is substantially dependent on the active participation of these healthcare professionals [18]. However, a systematic survey conducted by Chinese physicians focused on the diagnosis and patient management of type 1 diabetes (T1D) revealed that inadequate disease control in Chinese T1D patients can be attributed to ineffective therapeutic strategies prescribed by physicians [19]. Moreover, many medical professionals in various clinical settings exhibit limited knowledge and skepticism regarding the implementation and utilization of these systems [20, 21]. This knowledge gap and negative attitudes hinder the effective integration and utilization of information technology in diabetes management.

The knowledge, attitudes and practices (KAP) study, a research design, assesses the knowledge, attitudes, and behaviors pertaining to a specific health issue within a particular population. Through this approach, valuable insights are gained into the factors that influence decision-making and actions, aiding in the formulation of targeted interventions [22].

To address this issue, this study aimed to assess the knowledge, attitudes, and practices (KAP) among medical workers toward outpatient diabetes information platforms. By employing the KAP framework, this study investigated medical professionals’ levels of knowledge, attitudes, and practices.

## Methods

### Study design and participants

This cross-sectional study was conducted at the Department of Endocrinology of the First Hospital of Zhangjiakou between May 2023 and June 2023. This study included medical workers who engaged in frontline clinical practice. To maintain the focus on medical workers presently working within the clinical setting, rehired personnel were excluded. The study was ethically approved by the Ethics Committee of the First Hospital of Zhangjiakou. All participants signed informed consent prior to study, and informed consent was obtained from the participants.

### Questionnaire

The questionnaire was designed with reference to the related literature review and American Association of Clinical Endocrinology Clinical Practice Guideline: Developing a Diabetes Mellitus Comprehensive Care Plan-2022 Update [23, 24]. The first draft underwent revisions based on input from two senior experts, including a chief physician and a chief nurse. Subsequently, a small-scale pilot test was conducted (*n* = 60). The results of this pilot test yielded a Cronbach’s alpha coefficient value of 0.842, indicating a good internal consistency.

The final questionnaire was in Chinese and consisted of four dimensions: demographic information, knowledge, attitudes and practices. The demographic information was consisted of 10 items, and the knowledge, attitudes, and practices dimensions comprised 6, 14, and 9 items, respectively. The knowledge items were scored 1 point for a correct answer and 0 points for incorrect answers, resulting in a possible score range of 0–6. The attitudes items scored on a five-point Likert scale ranging from very positive (5 points) to very negative (1 point), with a possible score range of 14 to 70. The practices items also scored on a five-point Likert scale, ranging from always (5 points) to never (1 point), with a possible score range of 9 to 45.

The data were collected using an online questionnaire hosted on Sojump (http://www.sojump.com). The study was initiated by the First Hospital of Zhangjiakou, and the survey was distributed WeChat work contact groups, internal hospital forums, and web links in the form of web-based questionnaire. Participants included healthcare personnel associated with community units, medical management units, and external staff involved in long-term glycemic management, all linked with the First Hospital of Zhangjiakou. Additionally, two assistants were trained to provide online support to respondents for completing the questionnaire. To avoid duplicate entries, an IP restriction was enforced, allowing only one completion per unique IP address.

### Statistical methods

The statistical analysis was performed using STATA 17.0 (Stata Corporation, College Station, TX, USA). Continuous variables were presented as mean ± standard deviation (SD), while categorical variables were expressed as n (%). For continuous variables with a normal distribution, the t-test or ANOVA was applied. Pearson correlation analysis was used to assess the relationships among knowledge, attitudes, and practices. To examine the associations among knowledge, attitudes, and practices of medical workers towards the outpatient diabetes information platform, a structural equation model (SEM) was constructed, employing AMOS 24.0 (IBM, NY, United States). The SEM tested the following main hypotheses: (1) knowledge had direct effects on attitudes, (2) knowledge had direct effects on practices, and (3) attitudes had direct effects on practices. Model fit was evaluated using various indices, including CMIN/DF (Chi-square goodness-of-fit test/Degrees of Freedom), RMSEA (Root Mean Square Error of Approximation), IFI (Incremental Fixation Index), TLI (Tucker-Lewis Index), and CFI (Comparative Fixation Index). For multivariate logistic regression analysis, a cut-off value of 75% was applied, that means the threshold for sufficient knowledge, positive attitudes, and active practices were 4.5, 52.5 and 33.75 points respectively. A significance level of *P* < 0.05 was considered statistically significant.

## Results

A total of 685 questionnaires were collected. Among the participants, 603 (88.03%) were female, 432 (63.07%) work in a tertiary hospital, 548 (80.00%) have a bachelor degree, 270 (39.42%) of them work in the department of internal medicine and 315 (45.99%) of them received previous training on outpatient diabetes information platform (Table 1). Moreover, 350 (51.09%) of them have sufficient knowledge, 168 (24.53%) have positive attitudes and 395 (57.66%) have active practices (Table 2).

**Table 1:** Demographic characteristic and KAP scores

Variables	N (%)	Knowledge scores		Attitudes scores		Practices scores	
		Mean ± SD	*P*	Mean ± SD	P	Mean ± SD	*P*
**Total scores**	685	4.32 ± 1.27	-	56.76 ± 5.72	-	32.22 ± 8.42	-
**Gender** ^*^			0.300		0.869		0.002
Male	82 (11.97)	4.45 ± 1.29		56.66 ± 5.94		34.94 ± 8.03	
Female	603 (88.03)	4.30 ± 1.26		56.77 ± 5.69		31.85 ± 8.41	
**Age**	36.10 ± 8.18	-	-	-	-	-	-
**Type of hospital** ^**#**^			0.042		0.041		0.114
Primary Hospital	34 (4.96)	4.09 ± 1.31		55.91 ± 5.12		33.74 ± 7.02	
Secondary Hospital	219 (31.97)	4.17 ± 1.28		56.05 ± 5.34		31.31 ± 7.77	
Tertiary Hospital	432 (63.07)	4.41 ± 1.25		57.18 ± 5.91		32.56 ± 8.80	
**Highest Education** ^**#**^			<0.001		0.069		0.238
Junior college	122 (17.81)	3.89 ± 1.32		55.75 ± 6.58		33.32 ± 9.00	
Bachelor	548 (80.00)	4.39 ± 1.23		56.94 ± 5.52		31.94 ± 8.27	
Master and above	15 (2.19)	5.13 ± 1.13		58.27 ± 4.59		33.20 ± 8.72	
**Occupation** ^**#**^			<0.001		0.012		0.872
Doctor	134 (19.56)	4.75 ± 1.06		57.70 ± 4.87		32.49 ± 7.66	
Nurses	541 (78.98)	4.22 ± 1.29		56.60 ± 5.89		32.17 ± 8.63	
Community hospital staff	10 (1.46)	3.80 ± 1.55		52.80 ± 4.52		31.30 ± 6.53	
**Working year**	12.83 ± 8.17	-	-	-	-	-	-
**Professional title** ^**#**^			0.004		0.027		0.023
Junior	291 (42.48)	4.14 ± 1.34		56.21 ± 6.28		33.34 ± 8.58	
Intermediate	285 (41.61)	4.38 ± 1.21		56.78 ± 5.08		31.22 ± 8.50	
Associate senior	81 (11.82)	4.56 ± 1.16		58.09 ± 5.52		31.86 ± 7.56	
Senior	28 (4.09)	4.79 ± 1.00		58.32 ± 5.61		31.61 ± 7.11	
**Department** ^**#**^			0.154		0.309		0.009
Internal medicine	270 (39.42)	4.48 ± 1.18		57.28 ± 5.63		32.85 ± 7.99	
Surgery	144 (21.02)	4.17 ± 1.32		56.71 ± 5.53		32.17 ± 8.82	
Gynecologic	46 (6.72)	4.33 ± 1.08		56.52 ± 5.45		31.76 ± 7.58	
Pediatrics	38 (5.55)	4.21 ± 1.40		56.34 ± 6.84		27.26 ± 8.24	
Others (emergency department, intensive care medicine, nutrition, rehabilitation medicine, etc.), information department or equipment department	156 (22.77)	4.20 ± 1.32		55.95 ± 5.97		32.33 ± 9.03	
Department of medical technology	31 (4.53)	4.29 ± 1.51		57.32 ± 4.82		33.1 ± 6.67	
**Experience in diabetic management** ^*^			<0.001		<0.001		<0.001
Yes	544 (79.42)	4.44 ± 1.20		57.28 ± 5.45		32.81 ± 8.17	
No	141 (20.58)	3.84 ± 1.41		54.74 ± 6.28		29.93 ± 8.99	
**Previous training on outpatient diabetes platform** ^*^			0.285		<0.001		<0.001
Yes	315 (45.99)	4.37 ± 1.18		57.89 ± 5.09		35.17 ± 7.84	
No	370 (54.01)	4.27 ± 1.34		55.79 ± 6.04		29.70 ± 8.08	

*In* Table 1, the comparison between two groups was performed using the t-test, indicated by an asterisk (*). ANOVA was employed for comparisons among multiple groups, denoted by a hash symbol (#)

**Table 2:** Distribution of scores for knowledge, attitude, and practice

	N (%)
Knowledge score	
[0, 4.5]	335 (48.91)
(4.5, 6]	350 (51.09)
Attitude score	
[14, 52.50]	517 (75.47)
(52.50, 70]	168 (24.53)
Practice score	
[9, 33.75]	290 (42.34)
(33.75, 45]	395 (57.66)

*A cutoff value of 75% has been established, indicating that the thresholds for sufficient knowledge, positive attitudes, and active practices are 4.5, 52.5, and 33.75 points, respectively*

The three knowledge items with the highest accuracy rates were as follows: “The advantage of an outpatient diabetes information platform lies in its ability to effectively enhance blood glucose management for medical workers outside the hospital setting.“(K3) achieved an accuracy rate of 94.31%. “The outpatient diabetes information platform enables automated analysis of tasks and management processes.“(K5) achieved an accuracy rate of 89.20%. “Blood glucose measurement devices primarily consist of non-invasive, invasive, and minimally invasive methods.“(K2) achieved an accuracy rate of 76.35%. On the other hand, the three items with the lowest accuracy rates were: “Currently, the outpatient diabetes information platform does not enhance the reliability of the collected data.“(K6) achieved an accuracy rate of 36.50%. “The outpatient diabetes information platform does not contribute to reducing labor requirements or improving work efficiency“(K4) achieved an accuracy rate of 59.12%. “The source of blood glucose samples for determination is limited to capillary whole blood.“(K1) achieved an accuracy rate of 76.06% (Table 3).

**Table 3:** Knowledge

	Correctness N(%)
K1. The source of blood glucose samples for determination is limited to capillary whole blood. (wrong)	521 (76.06)
K2. Blood glucose measurement devices primarily consist of non-invasive, invasive, and minimally invasive methods. (right)	523 (76.35)
K3. The advantage of an outpatient diabetes information platform lies in its ability to effectively enhance blood glucose management for medical workers outside the hospital setting. (right)	646 (94.31)
K4. The outpatient diabetes information platform does not contribute to reducing labor requirements or improving work efficiency. (wrong)	405 (59.12)
K5. The outpatient diabetes information platform enables automated analysis of tasks and management processes. (right)	611 (89.20)
K6. Currently, the outpatient diabetes information platform does not enhance the reliability of the collected data. (wrong)	250 (36.50)

Medical workers have positive attitudes toward the outpatient diabetes information platform. 71.97% of the medical workers strongly agreed that the clinical intervention of the medical team in diabetic medical workers have an important impact on the level of blood glucose control and the incidence of complications in diabetic patients (A2). However, 31.68% of them strongly agreed that the outpatient diabetes information platform is inconvenient to use, and prefer traditional diabetes management methods (A14). Moreover, 32.99% of them strongly agreed with the notion that they experience role anxiety and distress while engaging in outpatient diabetes information management (A11) (Table 4).

**Table 4:** Attitudes

	Strongly agree	Agree	Neutral	Disagree	Strongly disagree
A1.The efficacy of out-of-hospital disease management plays a crucial role in determining the prognosis of diabetic patients.	482 (70.36)	177 (25.84)	23 (3.36)	1 (0.15)	2 (0.29)
A2. The clinical intervention of the medical team has an important impact on the level of blood glucose control and the incidence of complications in diabetic patients	493 (71.97)	166 (24.23)	24 (3.50)	0 (0.00)	2 (0.29)
A3. You recognize that the outpatient diabetes information platform can effectively stabilize the return rate of patients.	427 (62.34)	217 (31.68)	40 (5.84)	0 (0.00)	1 (0.15)
A4. You acknowledge that the information management system realizes the comprehensive control of lifestyle inside and outside the hospital	416 (60.73)	217 (31.68)	49 (7.15)	2 (0.29)	1 (0.15)
A5. You acknowledge that the outpatient diabetes information platform is of great help to medical workers to grasp the medical workers’ condition in a timely manner.	431 (62.92)	214 (31.24)	37 (5.40)	2 (0.29)	1 (0.15)
A6. You recognize the advantages of outpatient diabetes information platform in optimizing diagnosis and treatment plans.	420 (61.31)	223 (32.55)	41 (5.99)	0 (0.00)	1 (0.15)
A7. You recognize the important advantages of outpatient diabetes information platform over traditional telephone follow-up in clinical practice.	411 (60.00)	228 (33.28)	45 (6.57)	0 (0.00)	1 (0.15)
A8. You think that the outpatient diabetes information platform is an important part of industry, education and research, and its dynamic collection and processing of data can improve the scientific research level of the hospital.	411 (60.00)	229 (33.43)	41 (5.99)	2 (0.29)	2 (0.29)
A9. You are willing to participate in the relevant training and popular science lectures on the use of the outpatient diabetes information platform.	399 (58.25)	234 (34.16)	47 (6.86)	3 (0.44)	2 (0.29)
A10. You are willing to share with colleagues the experience of using the outpatient diabetes information platform and summarize the experience.	397 (57.96)	248 (36.20)	36 (5.26)	2 (0.29)	2 (0.29)
A11. In the process of outpatient diabetes information management, you have the problem of role anxiety and distress.	226 (32.99)	197 (28.76)	199 (29.05)	51 (7.45)	12 (1.75)
A12. You believe that your work attitudes is more susceptible to patient outcomes.	240 (35.04)	208 (30.36)	114 (16.64)	112 (16.35)	11 (1.61)
A13. You are confident that you can effectively use the outpatient diabetes information platform.	340 (49.64)	252 (36.79)	87 (12.70)	5 (0.73)	1 (0.15)
A14. You think that the outpatient diabetes information platform is inconvenient to use, and prefer traditional diabetes management methods.	217 (31.68)	137 (20.00)	149 (21.75)	167 (24.38)	15 (2.19)

Regarding the practices, 83.21% of the participants always/usually consciously study the relevant user manual of the outpatient diabetes information platform to understand the general workflow of the system (P1). Meanwhile, 79.86% of them claimed that they can update the latest guidelines and expert consensus on diabetes in a timely manner. However, only 32.41% of them confirmed that they always/usually use the outpatient diabetes information platform to detect the patient’s blood glucose level (P3) and 32.85% of them claimed that they always/usually push diagnosis and treatment opinions to medical workers through the outpatient diabetes information platform (P4). Additionally, 27.3% indicated that they never utilize the outpatient diabetes information platform for medication tracking (P5) (Table 5).

**Table 5:** Practices

	Always	Usually	Sometimes	Occasionally	Never
P1. You will consciously study the relevant user manual of the outpatient diabetes information platform to understand the general workflow of the system.	277 (40.44)	293 (42.77)	104 (15.18)	9 (1.31)	2 (0.29)
P2. You can update the latest guidelines and expert consensus on diabetes in a timely manner.	260 (37.96)	287 (41.90)	115 (16.79)	19 (2.77)	4 (0.58)
P3. How often you use the outpatient diabetes information platform to detect the patient’s blood glucose level.	129 (18.83)	93 (13.58)	128 (18.69)	148 (21.61)	187 (27.30)
P4. How often you push diagnosis and treatment opinions to medical workers through the outpatient diabetes information platform.	132 (19.27)	93 (13.58)	146 (21.31)	131 (19.12)	183 (26.72)
P5. The frequency of your medication tracking through the outpatient diabetes information platform.	134 (19.56)	89 (12.99)	140 (20.44)	135 (19.71)	187 (27.30)
P6. You will regularly evaluate the blood glucose compliance of medical workers in the use of the outpatient diabetes information platform.	183 (26.72)	245 (35.77)	158 (23.07)	51 (7.45)	48 (7.01)
P7. You will ask medical workers about their satisfaction with out-of-hospital diabetes management.	219 (31.97)	287 (41.90)	107 (15.62)	38 (5.55)	34 (4.96)
P8. You consciously review the experience of using the outpatient diabetes information platform and correct the shortcomings in the use process.	216 (31.53)	283 (41.31)	115 (16.79)	40 (5.84)	31 (4.53)
P9. In addition to clinical application, you will also consider the use value of the outpatient diabetes information platform in scientific research because of dynamic and reliable data.	242 (35.33)	309 (45.11)	90 (13.14)	21 (3.07)	23 (3.36)

Pearson correlation analysis showed that knowledge was positively correlated with attitudes (*r* = 0.397, *P <* 0.001), and attitudes were positively correlated with practices (*r* = 0.306, *P <* 0.001) (Table 6).

**Table 6:** Correlation analysis

	Knowledge	Attitudes	Practices
Knowledge	1		
Attitudes	0.397 (*p* < 0.001)	1	
Practices	0.059 (*p* = 0.122)	0.306 (*p* < 0.001)	1

Multivariate analysis showed that primary hospital (OR = 0.32, 95% CI: 0.14–0.71, *P =* 0.005), secondary hospital (OR = 0.48, 95% CI: 0.32–0.72, *P <* 0.001), doctor (OR = 2.44, 95% CI: 1.39–4.28, *P =* 0.002) were independently associated with sufficient knowledge. Knowledge (OR = 1.49, 95% CI: 1.29–1.73, *P <* 0.001), community hospital staff (OR = 0.21, 95% CI: 0.05–0.88, *P =* 0.032) were independently associated with positive attitudes. Attitudes (OR = 1.13, 95% CI: 1.09–1.17, *P <* 0.001), junior college (OR = 1.72, 95% CI: 1.07–2.77, *P =* 0.026) were independently associated with active practices (Table 7).

**Table 7:** Multivariate analysis

	OR (95% CI)	*P*
Knowledge		
**Type of hospital you work in**		
Primary Hospital	0.32 (0.14, 0.71)	0.005
Secondary Hospital	0.48 (0.32, 0.72)	< 0.001
Tertiary Hospital	Ref.	
**Educational level**		
Junior college	0.70 (0.45, 1.08)	0.109
Bachelor	Ref.	
Master	1.30 (0.33, 5.07)	0.708
**Type of occupation**		
Doctor	2.44 (1.39, 4.28)	0.002
Nurses	Ref.	
Community hospital staff	1.09 (0.27, 4.36)	0.905
**Professional title**		
Junior	Ref.	
Intermediate	1.24 (0.87, 1.79)	0.236
Associate senior	1.33 (0.73, 2.41)	0.346
Senior	2.25 (0.81, 6.20)	0.118
**Department**		
Internal medicine	Ref.	
Surgery	0.79 (0.50, 1.23)	0.291
Gynecologic	1.02 (0.53, 1.97)	0.957
Pediatrics	1.31 (0.63, 2.74)	0.472
Others (emergency department, intensive care medicine, nutrition, rehabilitation medicine, etc.), information department or equipment department	1.11 (0.72, 1.71)	0.647
Department of medical technology	1.92 (0.84, 4.40)	0.123
**experience in diabetic management**		
Yes	Ref.	
No	0.57 (0.37, 0.86)	0.008
**Attitudes**		
**Knowledge**	1.49 (1.29, 1.73)	< 0.001
**Type of occupation**		
Doctor	1.43 (0.82, 2.50)	0.209
Nurses	Ref.	
Community hospital staff	0.21 (0.05, 0.88)	0.032
**Department**		
Internal medicine	Ref.	
Surgery	0.85 (0.50, 1.42)	0.526
Gynecologic	0.76 (0.36, 1.61)	0.473
Pediatrics	1.41 (0.58, 3.44)	0.449
Others (emergency department, intensive care medicine, nutrition, rehabilitation medicine, etc.), information department or equipment department	0.73 (0.44, 1.21)	0.224
Department of medical technology	2.09 (0.73, 6.01)	0.172
**experience in diabetic management**		
Yes	Ref.	
No	0.55 (0.35, 0.87)	0.010
**Previous training on outpatient diabetes platform**		
Yes	Ref.	
No	0.51 (0.34, 0.75)	0.001
**Practices**		
**Knowledge**	0.86 (0.74, 1.00)	0.050
**Attitude**	1.13 (1.09, 1.17)	< 0.001
**Age**	0.95 (0.89, 1.01)	0.085
**Type of hospital you work in**		
Primary Hospital	1.46 (0.66, 3.25)	0.349
Secondary Hospital	1.00 (0.66, 1.53)	0.999
Tertiary Hospital	Ref.	
**Educational level**		
Junior college	1.72 (1.07, 2.77)	0.026
Bachelor	Ref.	
Master	2.88 (0.84, 9.89)	0.093
**Working year**	1.03 (0.97, 1.09)	0.341
**Professional title**		
Junior	Ref.	
Intermediate	0.75 (0.48, 1.19)	0.222
Associate senior	1.09 (0.51, 2.34)	0.826
Senior	1.05 (0.34, 3.27)	0.934
**Department**		
Internal medicine	Ref.	
Surgery	0.71 (0.44, 1.15)	0.168
Gynecologic	0.91 (0.45, 1.86)	0.802
Pediatrics	0.41 (0.16, 1.01)	0.053
Others (emergency department, intensive care medicine, nutrition, rehabilitation medicine, etc.), information department or equipment department	1.14 (0.72, 1.82)	0.571
Department of medical technology	1.14 (0.47, 2.75)	0.776
**experience in diabetic management**		
Yes	Ref.	
No	0.72 (0.45, 1.16)	0.176
**Previous training on outpatient diabetes platform**		
Yes	Ref.	
No	0.31 (0.22, 0.44)	< 0.001

**Fig. 1:**
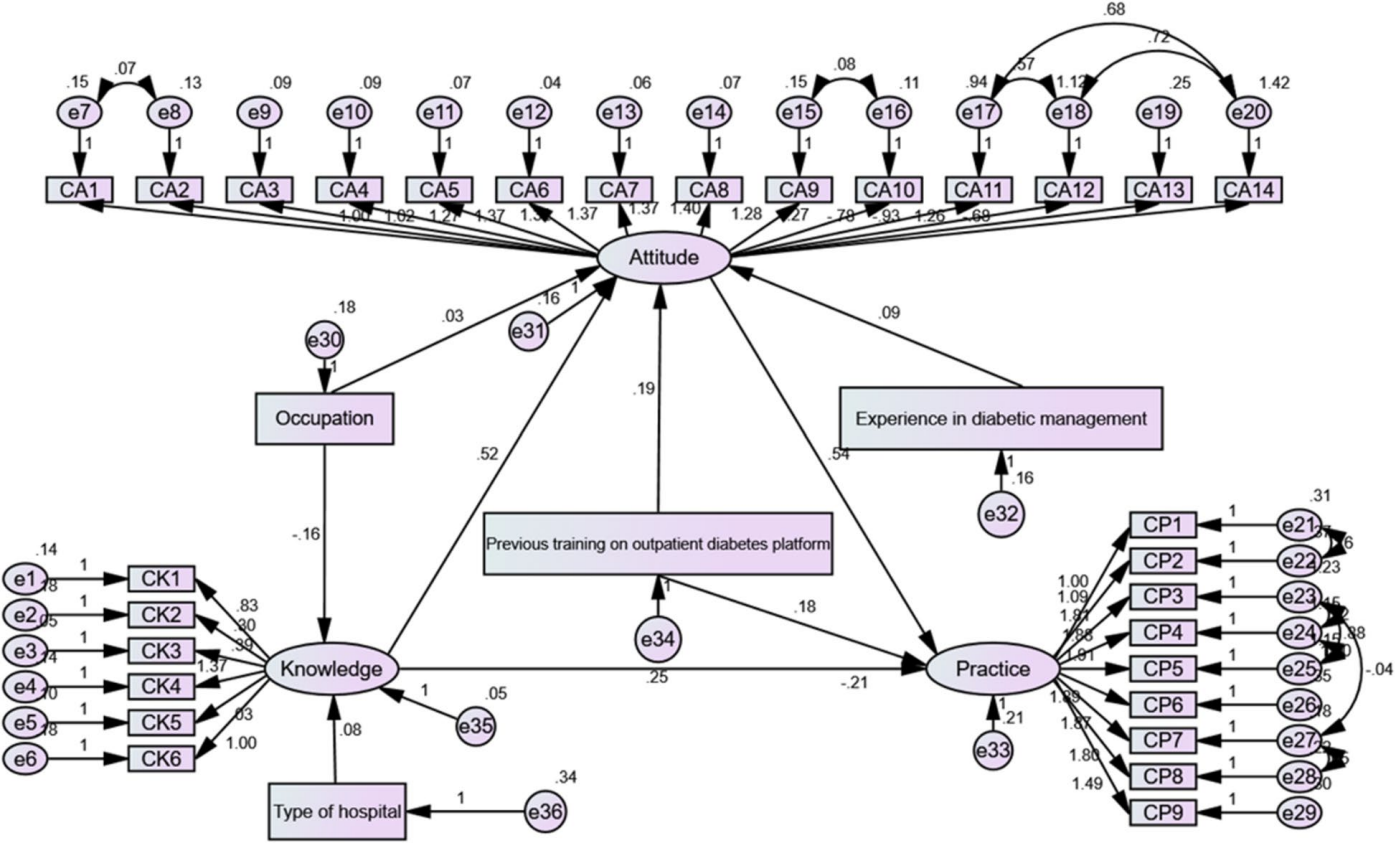
Structural equation modeling

The structural equation model demonstrated that knowledge exhibited a significant direct effect on attitudes (path coefficient = 0.521, *P <* 0.001), and attitudes showed a significant direct effect on practices (path coefficient = 0.542, *P <* 0.001). Moreover, the type of hospital had a direct impact on knowledge (path coefficient = 0.085, *P <* 0.001). Additionally, previous training on the outpatient diabetes platform had direct effects on attitudes (path coefficient = 0.191, *P <* 0.001) and practices (path coefficient = 0.184, *P <* 0.001). (Fig. 1; Table 8).

**Table 8:** Test results of the hypothesis

	Hypothesized paths			Path coefficient	*P* value
Hypothesis 1	Knowledge	<---	Occupation	-0.164	< 0.001
Hypothesis 2	Knowledge	<---	Type of hospital	0.085	< 0.001
Hypothesis 3	Attitudes	<---	Knowledge	0.521	< 0.001
Hypothesis 4	Attitudes	<---	Previous training on outpatient diabetes platform	0.191	< 0.001
Hypothesis 5	Attitudes	<---	Occupation	0.033	0.419
Hypothesis 6	Attitudes	<---	Experience in diabetic management	0.092	0.017
Hypothesis 7	Practices	<---	Attitudes	0.542	< 0.001
Hypothesis 8	Practices	<---	Knowledge	-0.207	0.047
Hypothesis 9	Practices	<---	Previous training on outpatient diabetes platform	0.184	< 0.001

The fitting index of the structural model (CMIN/DF = 3.599; RMSEA = 0.062; IFI = 0.928; TLI = 0.921; CFI = 0.928) outperformed the respective threshold value, signifying that the data fit the structural model satisfactorily (Table 9).

**Table 9:** Model fitness indices for the KAP structural equation model

Goodness-of-Fit Indices	Ideal standards	Measurement value
CMIN/DF	1–3 excellent, 3–5 good	3.599
RMSEA	< 0.08 good	0.062
IFT	> 0.8 good	0.928
TLI	> 0.8 good	0.921
CFI	> 0.8 good	0.928

*CMIN/DF *Chi-square fit statistics/degree of freedom, *RMSEA *Root mean square error of approximation, *IFI *Incremental fix index, *TLI *Tucker-Lewis index, *CFI *Comparative fix index

## Discussion

This study revealed that medical workers have insufficient knowledge, positive attitudes and inactive practices toward the outpatient diabetes information platform. Comprehensive training programs are needed to improve medical staff’s practices in this area.

In the previous study, a significant majority (94.31%) recognized the advantages of utilizing an outpatient diabetes information platform to effectively improve the blood glucose management of patients outside of the hospital setting. This recognition indicates a growing acknowledgment of the potential benefits such a platform can offer in supporting the management of diabetes. However, the study revealed that a smaller proportion (36.5%) believed that the outpatient diabetes information platform could currently enhance the reliability of the collected data. This finding highlights a perspective among certain participants regarding the platform’s ability to provide accurate and trustworthy data at present. One possibility is that the participants may have concerns about the accuracy and consistency of data input by patients themselves, as outpatient settings often rely on patient self-reporting [25]. However, a study demonstrated that the accuracy of self-reported diabetes was relatively high in a China study with a sample size of 1278 participants [26]. Therefore, it is crucial to enhance the knowledge of medical workers.

The attitudes dimension highlighted the generally positive attitudes towards outpatient diabetes information platforms, aligning with previous studies [27]. Most of the participants (71.97%) strongly agreed with the statement emphasizing the impact of medical team interventions on blood glucose control and complications in diabetic patients. However, it is noteworthy that a considerable proportion (31.68%) expressed agreement with the statement indicating inconvenience and a preference for traditional diabetes management methods, consistent with the findings of previous research that only a few diabetologist used diabetes apps to manage patients [20]. Additionally, 32.99% of the participants in the previous study strongly agreed that they experienced role anxiety and distress during the outpatient diabetes information management process, this align with other studies reflecting the emotional challenges faced by medical workers in this context [28]. Additionally, healthcare professionals who have previously received training on the outpatient diabetes platform demonstrated higher levels of attitudes, reflecting the significant role of training in enhancing the attitudes of medical staff towards the use of the outpatient diabetes platform (Tables 1 and 7). These findings underscore the importance of addressing concerns related to convenience, usability, and emotional well-being in order to ensure the successful implementation and acceptance of the platform among medical workers.

Furthermore, the participants actively engaged in familiarizing themselves with the platform’s operation, specifically in the process of acquiring knowledge. However, a notable proportion (27.3%) reported never tracking their medication frequency through the platform. This suggests a potential gap in utilizing the platform for medication tracking purposes. The reluctance of medical workers towards the platform can be attributed to various factors. A latent profile analysis has highlighted the significance of acknowledging the potential disassociation between individuals’ knowledge of new technologies and their negative attitudes towards medical artificial intelligence (AI) [21]. Similarly, a study on the ethical considerations for radiologists revealed that while these emerging technologies may be sensationalized to attract attention, they also have the potential to erode trust in the field if it becomes evident that the actual progress falls short of the promised advancements [29]. Moreover, the previous study revealed a significant association between previous training on the outpatient diabetes information platform and positive attitudes and good practices scores among participants. It indicates the importance of training programs in promoting effective utilization of the system. A study reported that standardized training on the management of diabetes led to improved knowledge of diagnosis and treatment among primary physicians, the screening rate for diabetes complications increased from 22.2% before training to 27.7% one year after training [30]. It is noteworthy that in this study, medical workers with junior professional titles achieved relatively higher practical scores (*P* = 0.023, Table 1). This finding may initially appear counterintuitive to conventional expectations. Typically, it is assumed that medical professionals with higher titles, due to their richer experience and expertise, would perform better in practical assessments. However, this situation could be attributed to several factors. Firstly, medical workers with senior titles might be preoccupied with managerial responsibilities or other advanced tasks, possibly limiting their time and focus to thoroughly understand and use the platform [31]. Moreover, medical workers with junior titles might exhibit a more positive attitude towards new technologies and processes, showing a willingness to experiment with and implement a novel outpatient diabetes information platform [32, 33]. Furthermore, healthcare professionals with prior training on the outpatient diabetes platform exhibited higher levels of practice, consistent with the findings regarding attitude levels in this study. This underscores the crucial role that training plays in improving the practices of medical staff in the utilization of the outpatient diabetes platform (Tables 1 and 7). Therefore, comprehensive training programs are needed to improve medical staff’s practices in this area.

There are several limitations to consider in this study. Firstly, the study was conducted in a specific region, which may limit the generalizability of the findings to other settings. Additionally, the sample size of this study might restrict the representativeness of the study population, thus potentially contributing to the non-statistically significant results observed in the Pearson analysis and SEM, which examined the relationship between knowledge and attitudes. It is imperative to undertake further research across various regions to analyze the relationship between KAP levels and the regional disparities in medical and economic standards. This expanded scope of study will provide a more comprehensive understanding of the interplay between these variables.

Nevertheless, this study highlights the need for improved knowledge, attitudes, and practices among medical workers toward the outpatient diabetes information platform. The findings indicate that training programs and interventions should be implemented to address the gaps and enhance the utilization of these systems.

### Supplementary Information

**Supplementary Material 1.**

## Data Availability

The datasets used and/or analysed during the current study are available from the corresponding author on reasonable request.
