# Supplementary material for: Knowledge, attitudes and practices among medical workers toward outpatient diabetes information platform
Source: BMC Health Serv Res. 2024 Mar 12;24:328. doi: 10.1186/s12913-024-10711-y (PMC10935856; doi:10.1186/s12913-024-10711-y)
Supplement: Supplementary file 1 — Supplementary Material 1. [file 12913_2024_10711_MOESM1_ESM.docx]

| **Part I Demographic information** | |
| --- | --- |
| 1. **Gender** | a.Male  b.Female |
| 1. **Age (years)：** | _____ |
| 1. **Type of hospital：** | a.Primary Hospital  b.Secondary Hospital  c.Tertiary Hospitals |
| 1. **Highest Education** | a.Junior college  b.Bachelor  c.Master and above |
| 1. **Occupation：** | a.Doctor  b.Nurses  c.Community hospital staff |
| 1. **Working year** | _____ |
| 1. **Professional title：** | a.Junior  b.Intermediate  c.Associate senior  d.Senior |
| 1. **Department：** | a.Internal medicine  b.Surgery  c.Gynecologic  d.Pediatrics  e.Others (emergency department, intensive care medicine, nutrition, rehabilitation medicine, etc.), information department or equipment department  f.Department of medical technology |
| 1. **Experience in diabetic management** | a.Yes  b.No |
| 1. **Previous training on outpatient diabetes platform** | a.Yes  b.No |

| **Part II Knowledge**   \| K1. The source of blood glucose samples for determination is limited to capillary whole blood. \| a.Yes \| b.No \| \| --- \| --- \| --- \| \| K2. Blood glucose measurement devices primarily consist of non-invasive, invasive, and minimally invasive methods. \| a.Yes \| b.No \| \| K3. The advantage of an outpatient diabetes information platform lies in its ability to effectively enhance blood glucose management for medical workers outside the hospital setting. \| a.Yes \| b.No \| \| K4. The outpatient diabetes information platform does not contribute to reducing labor requirements or improving work efficiency. \| a.Yes \| b.No \| \| K5. The outpatient diabetes information platform enables automated analysis of tasks and management processes. \| a.Yes \| b.No \| \| K6. Currently, the outpatient diabetes information platform does not enhance the reliability of the collected data. \| a.Yes \| b.No \| |
| --- | --- | --- | --- | --- | --- | --- | --- | --- | --- | --- | --- | --- | --- | --- | --- | --- | --- | --- |

| **Part III Attitude** | | | | | |
| --- | --- | --- | --- | --- | --- |
| A1.The efficacy of out-of-hospital disease management plays a crucial role in determining the prognosis of diabetic patients. | a.Strongly Agree | b.Agree | c.Neutral | d.Disagree | e.Strongly disagree |
| A2. The clinical intervention of the medical team has an important impact on the level of blood glucose control and the incidence of complications in diabetic patients | a.Strongly Agree | b.Agree | c.Neutral | d.Disagree | e.Strongly disagree |
| A3. You recognize that the outpatient diabetes information platform can effectively stabilize the return rate of patients. | a.Strongly Agree | b.Agree | c.Neutral | d.Disagree | e.Strongly disagree |
| A4. You acknowledge that the information management system realizes the comprehensive control of lifestyle inside and outside the hospital | a.Strongly Agree | b.Agree | c.Neutral | d.Disagree | e.Strongly disagree |
| A5. You acknowledge that the outpatient diabetes information platform is of great help to medical workers to grasp the medical workers' condition in a timely manner. | a.Strongly Agree | b.Agree | c.Neutral | d.Disagree | e.Strongly disagree |
| A6. You recognize the advantages of outpatient diabetes information platform in optimizing diagnosis and treatment plans. | a.Strongly Agree | b.Agree | c.Neutral | d.Disagree | e.Strongly disagree |
| A7. You recognize the important advantages of outpatient diabetes information platform over traditional telephone follow-up in clinical practice. | a.Strongly Agree | b.Agree | c.Neutral | d.Disagree | e.Strongly disagree |
| A8. You think that the outpatient diabetes information platform is an important part of industry, education and research, and its dynamic collection and processing of data can improve the scientific research level of the hospital. | a.Strongly Agree | b.Agree | c.Neutral | d.Disagree | e.Strongly disagree |
| A9. You are willing to participate in the relevant training and popular science lectures on the use of the outpatient diabetes information platform. | a.Strongly Agree | b.Agree | c.Neutral | d.Disagree | e.Strongly disagree |
| A10. You are willing to share with colleagues the experience of using the outpatient diabetes information platform and summarize the experience. | a.Strongly Agree | b.Agree | c.Neutral | d.Disagree | e.Strongly disagree |
| A11. In the process of outpatient diabetes information management, you have the problem of role anxiety and distress. | a.Strongly Agree | b.Agree | c.Neutral | d.Disagree | e.Strongly disagree |
| A12. You believe that your work attitudes is more susceptible to patient outcomes. | a.Strongly Agree | b.Agree | c.Neutral | d.Disagree | e.Strongly disagree |
| A13. You are confident that you can effectively use the outpatient diabetes information platform. | a.Strongly Agree | b.Agree | c.Neutral | d.Disagree | e.Strongly disagree |
| A14. You think that the outpatient diabetes information platform is inconvenient to use, and prefer traditional diabetes management methods. | a.Strongly Agree | b.Agree | c.Neutral | d.Disagree | e.Strongly disagree |

| **Part IV Practice** |
| --- |
| P1. You will consciously study the relevant user manual of the outpatient diabetes information platform to understand the general workflow of the system.  a. Always ( frequency > 6 times in the past 2 months)  b. Often ( frequency 5-6 times in the past 2 months)  c. Sometimes ( frequency 3-4 times in the past 2 months)  d. Occasionally ( frequency 1-2 times in the past 2 months)  e. Never ( frequency 0 times in the past 2 months) |
| P2. You can update the latest guidelines and expert consensus on diabetes in a timely manner.  a. Always ( frequency > 6 times in the past 2 months)  b. Often ( frequency 5-6 times in the past 2 months)  c. Sometimes ( frequency 3-4 times in the past 2 months)  d. Occasionally ( frequency 1-2 times in the past 2 months)  e. Never ( frequency 0 times in the past 2 months) |
| P3. How often you use the outpatient diabetes information platform to detect the patient's blood glucose level.  a. Always ( frequency > 6 times in the past 2 months)  b. Often ( frequency 5-6 times in the past 2 months)  c. Sometimes ( frequency 3-4 times in the past 2 months)  d. Occasionally ( frequency 1-2 times in the past 2 months)  e. Never ( frequency 0 times in the past 2 months) |
| P4. How often you push diagnosis and treatment opinions to medical workers through the outpatient diabetes information platform.  a. Always ( frequency > 6 times in the past 2 months)  b. Often ( frequency 5-6 times in the past 2 months)  c. Sometimes ( frequency 3-4 times in the past 2 months)  d. Occasionally ( frequency 1-2 times in the past 2 months)  e. Never ( frequency 0 times in the past 2 months) |
| P5. The frequency of your medication tracking through the outpatient diabetes information platform.  a. Always ( frequency > 6 times in the past 2 months)  b. Often ( frequency 5-6 times in the past 2 months)  c. Sometimes ( frequency 3-4 times in the past 2 months)  d. Occasionally ( frequency 1-2 times in the past 2 months)  e. Never ( frequency 0 times in the past 2 months) |
| P6. You will regularly evaluate the blood glucose compliance of medical workers in the use of the outpatient diabetes information platform.  a. Always ( frequency > 6 times in the past 2 months)  b. Often ( frequency 5-6 times in the past 2 months)  c. Sometimes ( frequency 3-4 times in the past 2 months)  d. Occasionally ( frequency 1-2 times in the past 2 months)  e. Never ( frequency 0 times in the past 2 months) |
| P7. You will ask medical workers about their satisfaction with out-of-hospital diabetes management.  a. Always ( frequency > 6 times in the past 2 months)  b. Often ( frequency 5-6 times in the past 2 months)  c. Sometimes ( frequency 3-4 times in the past 2 months)  d. Occasionally ( frequency 1-2 times in the past 2 months)  e. Never ( frequency 0 times in the past 2 months) |
| P8. You consciously review the experience of using the outpatient diabetes information platform and correct the shortcomings in the use process.  a. Always ( frequency > 6 times in the past 2 months)  b. Often ( frequency 5-6 times in the past 2 months)  c. Sometimes ( frequency 3-4 times in the past 2 months)  d. Occasionally ( frequency 1-2 times in the past 2 months)  e. Never ( frequency 0 times in the past 2 months) |
| P9. In addition to clinical application, you will also consider the use value of the outpatient diabetes information platform in scientific research because of dynamic and reliable data.  a. Always ( frequency > 6 times in the past 2 months)  b. Often ( frequency 5-6 times in the past 2 months)  c. Sometimes ( frequency 3-4 times in the past 2 months)  d. Occasionally ( frequency 1-2 times in the past 2 months)  e. Never ( frequency 0 times in the past 2 months) |
